# EUS-Guided Biopsy with a Novel Puncture Biopsy Forceps Needle—Feasibility Study

**DOI:** 10.3390/diagnostics11091638

**Published:** 2021-09-07

**Authors:** Geke Litjens, Christian Gerges, Yogesh M. Shastri, Piyush Somani, Torsten Beyna, Horst Neuhaus, Cornelis J. H. M. van Laarhoven, Mathias Prokop, Peter D. Siersema, John J. Hermans, Erwin J. M. van Geenen

**Affiliations:** 1 Department of Medical Imaging, Radboud Institute for Health Sciences, Radboud University Medical Center, 6500 HB Nijmegen, The Netherlands; G.Litjens@radboudumc.nl (G.L.); Mathias.Prokop@radboudumc.nl (M.P.); John.Hermans@radboudumc.nl (J.J.H.); 2 Department of General Internal Medicine and Gastroenterology, Evangelisches Krankenhaus Düsseldorf, 40217 Düsseldorf, Germany; Christian.Gerges@evk-duesseldorf.de (C.G.); Torsten.Beyna@evk-duesseldorf.de (T.B.); Horst.Neuhaus@evk-duesseldorf.de (H.N.); 3 Department of Gastroenterology and Hepatology, NMC Specialty Hospital, Abu Dhabi 6222, United Arab Emirates; drshastri@gmail.com; 4 Department of Gastroenterology, NMC Royal Hospital Sharjah, Sharjah 3499, United Arab Emirates; dr_piyushsomani@yahoo.co.in; 5 Department of Gastroenterology, NMC Specialty Hospital, Dubai 7832, United Arab Emirates; 6 Department of Surgery, Radboud Institute for Health Sciences, Radboud University Medical Center, 6500 HB Nijmegen, The Netherlands; Kees.vanLaarhoven@radboudumc.nl; 7 Department of Gastroenterology and Hepatology, Radboud Institute for Molecular Life Sciences, Radboud University Medical Center, 6500 HB Nijmegen, The Netherlands; Peter.Siersema@radboudumc.nl

**Keywords:** endoscopic ultrasound, image-guided biopsy, biopsy, EUS-guided tissue acquisition, diagnostic accuracy

## Abstract

Endoscopic ultrasound (EUS) with fine needle aspiration (FNA) or biopsy (FNB) to diagnose lesions in the gastrointestinal tract is common. Demand for histology sampling to identify treatment-specific targets is increasing. Various core biopsy FNB needles to obtain tissue for histology are currently available, however, with variable (37–97%) histology yields. In this multicenter study, we evaluated performance, safety, and user experience of a novel device (the puncture biopsy forceps (PBF) needle). Twenty-four procedures with the PBF needle were performed in 24 patients with a suspected pancreatic lesion (*n* = 10), subepithelial lesion (*n* = 10), lymph node (*n* = 3), or pararectal mass (*n* = 1). In 20/24 (83%) procedures, the PBF needle yielded sufficient material for interpretation (sample adequacy). In 17/24 (71%), a correct diagnosis was made with the material from the PBF needle (diagnostic accuracy). All participating endoscopists experienced a learning curve. (Per)procedural technical issues occurred in four cases (17%), but there were no adverse events. The PBF needle is a safe and potentially useful device to obtain an EUS-guided biopsy specimen. As the design of the PBF needle is different to core biopsy FNB needles, specific training will likely further improve the performance of the PBF needle. Furthermore, the design of the needle needs further improvement to make it more robust in clinical practice.

## 1. Introduction

Endoscopic ultrasound (EUS) is nowadays increasingly used for the evaluation of lesions in the gastrointestinal tract [1]. EUS can be combined with tissue acquisition to further characterize these lesions. EUS with fine needle aspiration (FNA) can be used to obtain the cytology of lesions in solid organs, lymph nodes, and tumors of the gastrointestinal tract. The performance of FNA needles for diagnosing pancreatic lesions is relatively good with a pooled sensitivity in the range of 85–89% [2]. However, with the rapid development of precision medicine in oncology there is an increasing demand for tissue histology sampling instead of cytology, for additional immunohistochemistry and analysis of molecular biomarkers to identify treatment targets [3,4]. Furthermore, diagnosing non-pancreatic lesions often requires samples with a high cellularity and assessment of lesion architecture and morphology [5,6]. For subepithelial masses and lymph nodes, FNA performance varies with a diagnostic accuracy of 46–98% and 65–100%, respectively [2].

In the last decade, several fine needle biopsy (FNB) needles have been developed. Most needles are ‘core biopsy’ style needles. However, the yield of obtaining a satisfactory histology result with these FNB needles is highly variable, with histological core procurement in the range of 37–97% [7,8,9,10,11].

**Figure 1:**
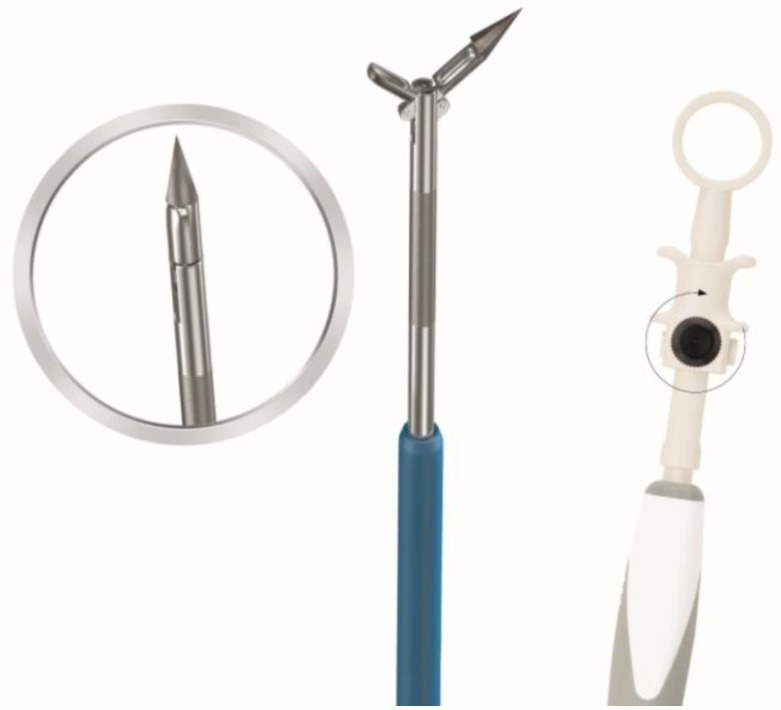
The puncture biopsy forceps (PBF needle) with a forceps mechanism in the needle tip, the round arrow indicates the locking screw. Image provided by MTW Endoskopie Manufaktur.

In an effort to increase the histology yield for EUS-guided biopsies, a novel device was recently developed: the nitinol 19-gauge puncture biopsy forceps (PBF needle, MTW (Medizinisch Technische Werkstätte) Endoskopie Manufaktur, Wesel, Germany, Figure 1) [12]. The forceps diameter is 1.1 mm, the tube diameter is 1.95 mm, the tube length is 142.2 cm, and the maximum forceps outlet is 7.5 cm. The needle has a different design compared to other FNB needles recently developed. Instead of obtaining a histological core, it has a forceps mechanism, which can be opened once inside the lesion and subsequently closed to grasp a tissue fragment. The needle design was improved following feedback after the use of the first version [12]. The needle tip is now manufactured from nitinol making the needle flexible without losing its shape during puncturing; a safety locking screw was added for fixing the branches, and ultrasound marking was applied to the tip to improve visibility. In this study, we present our experiences and initial results with the latest design of the PBF needle.

## 2. Materials and Methods

We performed a multicenter retrospective cohort study to evaluate the performance, safety, and user experience of EUS-guided biopsy with the PBF needle (MTW Endoskopie Manufaktur, Wesel, Germany). The Medical Ethics Committee of the Radboud university medical center approved this study (Approved code 2020-6834, Approved date 16 July 2020). All cases in which EUS-guided biopsy with the PBF needle was performed were identified and included in this study. The four participating centers included: Radboud university medical center, Nijmegen, the Netherlands (RUMC, coordinating center); Evangelisches Krankenhaus, Düsseldorf, Germany (EVK); New Medical Center Specialty Hospital, Abu Dhabi, United Arab Emirates (NMC); and Thumbay Hospital, Dubai, United Arab Emirates (THD).

### 2.1. EUS-Procedure and Tissue Acquisition

A total of five endoscopists used the PBF needle in the period January 2018 until March 2020 (two endoscopists from EVK and one from each of the other centers). The participating endoscopists were all experienced in EUS-guided biopsy, with 6–12 years of experience with EUS, and each performing 120–300 EUS procedures/year. The EUS procedure was performed according to local clinical protocols and the choice of needles and number of passes (number of times the needle was punctured into the lesion to collect a specimen) was at the endoscopists discretion. Patients were informed about the use of the new needle before the procedure. The endoscopists documented in which cases they used the PBF needle and clinical data was retrieved retrospectively. In most cases, additionally to the PBF needle another FNA or FNB needle was used during the same procedure. The PBF and FNA/B histopathologic specimens were analyzed separately.

### 2.2. Outcome Parameters and Definitions

The primary outcome was the sample adequacy of the PBF needle, defined as the proportion of cases in which biopsy with the PBF needle led to sufficient histological material for interpretation, as reported by the pathologist. Secondary outcomes were: diagnostic accuracy, defined as the proportion of cases in which biopsy with the PBF needle yielded a correct pathological diagnosis, which was correlated to the definitive diagnosis as confirmed by resection or follow-up imaging; safety; and complications. Furthermore, (per)procedural technical issues where reported, and finally, the user experience was analyzed, for which all participating endoscopists completed a questionnaire after they had performed all procedures. The questionnaire consisted of eight aspects about the use of the PBF needle, which were scored from 1–10, and four statements about their experience with the needle, which were answered with yes or no. Several general aspects of EUS-needles, such as visibility on ultrasound, ease of puncturing, and specific needle properties for the PBF needle, such as the forceps mechanism, were included in the questionnaire. The full questionnaire can be found in Supplementary.

### 2.3. Statistics

Anonymized data was shared with the coordinating center for analysis. Continuous variables were recorded as means or medians with standard deviation or range. Categorical variables were recorded as frequencies with percentages. We decided not to use sensitivity and specificity as outcome measures, because different lesion types were included, both malignant and benign. A comparison of the technical performance by lesion location was performed using the Fisher-Freeman-Halton exact test. Analyses were performed using SPSS version 25 (IBM Corp., Armonk, New York, NY, USA) for Windows.

## 3. Results

A total of 24 procedures were performed with the PBF needle between January 2018 and March 2020. The majority of patients were male (17, 71%) and the mean age was 56 (range 34–78) years of age. Most procedures (*n* = 10) were performed at NMC, followed by EVK with 6 procedures, and RUMC and THD, where 4 procedures each were performed. Biopsies were performed on 10 pancreatic lesions (41.7%), 10 subepithelial lesions (41.7%; esophageal *n* = 1, gastroesophageal junction *n* = 3, gastric *n* = 4, duodenal *n* = 1, and small bowel *n* = 1), three lymph nodes (12.5%; mediastinal *n* = 2 and peripancreatic *n* = 1), and one pararectal mass (4.2%). The median number of passes with the PBF needle in each case was three (range 1–7). In 22 cases (92%) another needle was additionally used during the same EUS procedure: FNA (*n* = 14), FNB (*n*= 7), or both FNA and FNB (*n*= 1). The final diagnosis was pancreatic ductal adenocarcinoma (PDAC) in seven cases, leiomyoma in six cases, benign (reactive) lymph nodes in three cases, pancreatitis in two cases, linitis plastica in two cases, and the remaining four cases all had a different diagnosis. Table 1 displays the patient and procedure characteristics.

### 3.1. Sample Adequacy

The PBF needle was successfully punctured in the targeted lesion in all cases. However, in one case, a pancreatic lesion, the needle could not be opened once inside the lesion; therefore, in this case no tissue could be obtained with the PBF needle. This was probably caused by fibrotic tissue that was present. Of the remaining 23 cases, in one case there were no tissue fragments present at pathological examination, in two cases there was tissue present but not enough to perform pathological examination, but in 20 cases adequate material for pathological examination was successfully obtained. This leads to a sample adequacy of the PBF needle of 83% (20/24; Table 2). The sample adequacy was not significantly different between non-pancreatic versus pancreatic lesions (*p* = 0.272; Table 3).

### 3.2. Diagnostic Accuracy

The tissue obtained with the PBF needle led to a correct diagnosis in 17 cases, therefore the diagnostic accuracy of the PBF needle was 71% (17/24). Of the seven cases without a correct diagnosis with the PBF needle, four of those cases were because no or not enough tissue was obtained (as described above), and in three cases sufficient tissue was obtained but the correct diagnosis could not be made on the tissue. In two of these cases not enough pancreatic or submucosal tissue, respectively, was obtained for a correct diagnosis. In a case of linitis plastica the tissue was suspicious for lymphoid lymphoma but no definitive diagnosis could be made. In 10 cases both the tissue obtained with the PBF needle as well as with the additionally used FNA or FNB needle was diagnostic. In six cases the PBF needle led to a correct diagnosis, whereas this was not the case with FNA (2 cases) or FNB (4 cases), respectively. In one case the PBF needle led to a correct diagnosis and no other needle was used. In four of seven cases without a correct diagnosis with the PBF needle, the correct diagnosis was made with FNA (3 cases) or FNB (1 case), in two cases cytology obtained with FNA during the same session was also incorrect, and in one case no other FNA or FNB needle was used, and a final diagnosis of the lesion was made after endoscopic resection. These results are displayed in Table 2. The diagnostic accuracy was not significantly different between non-pancreatic versus pancreatic lesions (*p* = 0.393; Table 3).

### 3.3. Safety and Technical Use

There were no procedure-related adverse events observed in these 24 cases. In four cases (17%) (per)procedural technical issues occurred. In two cases the spring mechanism of the needle broke causing the forceps mechanism in the tip of the needle to malfunction. In both of these cases at least one biopsy was taken before this mechanism broke. In one case with a pancreatic lesion the needle could not be opened once inside the lesion (see also above). Finally, in one case the handle of the needle broke after three successful passes were already performed. The (per)procedural technical issues did not cause any harm to the patients involved.

### 3.4. User Experience

The average endoscopist experience score of all eight features from the questionnaire was 6 on a scale of 1–10 (range 5.3–7.3; Table 4). The highest score was for the needle visibility on US imaging; this was scored with a 7.2 on average (range 6–8). The opinion of the endoscopists about the tissue yield had the widest range (3–8) with an average score of 6.4. Although the PBF needle has a flexible nitinol tip, which allows the needle tip to bend while returning to its original position once straightened again, the endoscopists rated the use of the needle in a bended scope position with a mean score of 4.2 (range 3–6).

The outcome of the four user experience statements was as follows. Firstly, all endoscopists stated that they experienced a learning curve, especially for the opening and closing mechanism of the forceps and the technique to obtain a high tissue amount (pushing the needle inwards after opening of the forceps). Secondly, 3/5 endoscopists declared that they would also use the needle in the future. Thirdly, one endoscopist would recommend the needle to his colleagues and three would recommend the needle under certain conditions of use, such as only for cases with large submucosal lesions or after further improvement of the needle design had occurred. Finally, 3/5 endoscopists reported technical problems with the needle. The endoscopists did not receive specific training before using the PBF needle. The technical problems have already been described above.

## 4. Example Case

**Figure 2:**
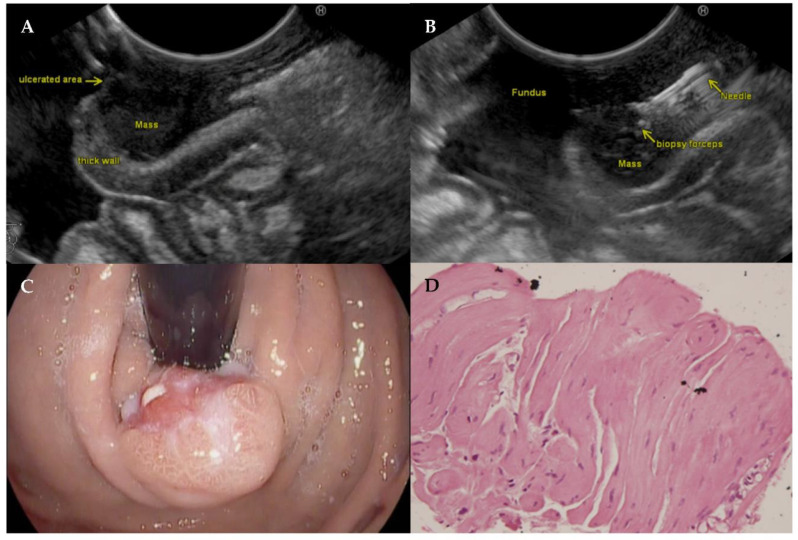
EUS image of subepithelial leiomyoma at gastroesophageal junction (**A**). EUS image of the leiomyoma with the opened puncture biopsy forceps needle inside (**B**). Gastroduodenal endoscopy of the subepithelial leiomyoma at the gastroesophageal junction (**C**). Hematoxylin and eosin staining of the histologic tissue fragment of the biopsy, magnification 400× (**D**).

A 34-year-old male with reflux symptoms and an ulcerated polypoidal subepithelial lesion at the gastroesophageal (GE) junction was scheduled for EUS with biopsy. EUS showed a hypoechoic lesion arising from the muscularis propria involving all layers of the GE junction. A histologic tissue fragment was obtained with the PBF needle which showed a leiomyoma at histologic analysis. Figure 2 displays the EUS, endoscopy, and histology images.

## 5. Discussion

This multicenter cohort study evaluated the sample adequacy, diagnostic accuracy, safety, and user experience of a novel needle with biopsy forceps for EUS-guided tissue acquisition in 24 cases. The sample adequacy of the PBF needle was 83%. Although there were some (per)procedural technical issues, the diagnostic accuracy was 71% and no adverse events were observed. In six procedures the PBF needle led to a correct diagnosis with FNA or FNB not being able to provide a diagnosis, compared to four procedures in which FNA or FNB led to a correct diagnosis with the PBF needle not being diagnostic. The involved endoscopists scored the use of the needle on several features in a questionnaire with an average score of 6 out of 10.

Current guidelines do not favor FNA or FNB for tissue acquisition of lesions in the gastrointestinal tract, but more and more data now suggest an advantage of FNB over FNA as it allows the collection of a histological specimen, opposed to cytology alone [6,13,14]. The sample adequacy of the PBF needle found in this study (83%) is comparable to the results of several FNB needles for histological tissue acquisition, with results for sample adequacy in the range of 70–95% [7,9,11].

The success rates of FNA and/or FNB needles in obtaining adequate tissue vary significantly according to the lesion type and location. For example, for subepithelial lesions the pooled diagnostic rate reported in a large meta-analysis was 60% for FNA/FNB [15]. In our study, we obtained a sample adequacy of 90% (9/10) for subepithelial lesions with the PBF needle. No statistical differences in sample adequacies of the PBF needle between the different lesion locations were seen. This is likely explained by the low number of cases per lesion location.

For solid pancreatic masses, a recent network meta-analysis comparing needle sizes and designs (FNA versus FNB) showed no differences in the used sampling technique [16]. The diagnostic accuracy for the PBF needle (71%) is in the lower range of previously reported diagnostic accuracies for FNA/FNB (63–92%) [14,17,18,19]. This was however mainly due to (per)procedural technical problems with the handle or the opening and closing spring mechanism of the PBF needle. Technical improvements are therefore needed, enabling the use of the PBF needle in fibrotic lesions as well as in solid pancreatic cancer and to make it as safe and reliable as FNA/FNB needles [6]. The PBF needle is a technically more demanding device compared to FNA/FNB needles but has the potential for a higher tissue yield. In addition, the learning curve reported by all endoscopists of this novel device may also have influenced the results. Switching from an FNA needle to an FNB needle requires only a slight adjustment during the biopsy procedure, as the handling is similar, as opposed to operating the forceps mechanism of the PBF needle. Furthermore, not only the endoscopist, but also the assisting nurse or technician could benefit from training on the technique of operating the opening and closing mechanism of the PBF needle, while the role of the assistant is negligible during FNA/FNB. Despite the technical issues and limited experience of the endoscopists with the PBF needle in our study, the sample adequacy rate of 83% shows the potential of a significantly higher performance once these difficulties have been overcome. Finally, the device seemed to be safe with no adverse events during this study.

Our study has some limitations. It was a retrospective study, in which the number of passes and the use of another FNA/FNB needle were not standardized. This could have resulted in selection bias as the choice for additional use and type of needle was at the discretion of the endoscopist. However, if there was selection bias, it is likely that this has resulted in selection of more challenging cases and those in which histology was required. Furthermore, the specimens were not evaluated by a central pathologist. Unfortunately, we were not able to quantify the number of histology specimens obtained with the PBF needle in this study. We hypothesize that pancreatic lesions were more challenging to biopsy with the PBF needle than subepithelial lesions, particularly in the beginning. Due to a heterogeneous patient group with different lesion types, both pancreatic (*n* = 10) and non-pancreatic (subepithelial *n* = 10, lymph node *n* = 3, pararectal mass *n* = 1), being included in this study, this difference in learning curve has likely affected the results. While the number of pancreatic and subepithelial lesions allows at least a certain interpretation of the results in this particular group, the other entities (lymph nodes and pararectal mass) are underrepresented in a way that an adequate subgroup analysis is not possible.

Identified strengths are that it was a study in a real life setting of five endoscopists in four different centers, with a direct comparison of an alternative needle in almost all cases. Additionally, a structured questionnaire, which was answered by all participating endoscopists after all procedures were finished, was used to assess user experience.

## 6. Conclusions

In conclusion, we have shown that the PBF needle is a safe and potentially promising but technically demanding device. Adequate histologic tissue samples are needed for genomic medicine, and this is an emerging field in EUS-FNB. The PBF needle is potentially suitable for precision oncology. We believe that improvements in robustness and proctoring of the needle are needed before being used further in clinical practice. In addition, it needs to be established for which lesion type the PBF needle is most effective. A comparison of an improved PBF needle design versus other FNB needles on histology yield is needed, preferably, in a well-designed randomized controlled trial.

## Figures and Tables

**Table 1:** Patient and procedure characteristics.

Characteristics	
Male gender, *n* (%)	17 (71%),
Age, median (range)	57 (range 34–78)
Center of biopsy	
NMC	10
EVK	6
RUMC	4
THD	4
Target lesion	
Pancreas	10
Subepithelial lesion	10
Lymph node	3
Pararectal mass	1
Number of passes with MTW needle, median (range)	3 (1–7)
Use of another FNA/FNB needle, *n* (%)	22 (92%)
FNA	14
FNB	7
both FNA and FNB	1
Final diagnosis, *n*	
PDAC	7
Leiomyoma	6
Benign LN	3
Pancreatitis	2
Linitis plastica	2
NET	1
GIST	1
Lymphoma	1
Fibrosis	1

NMC: New Medical Center Specialty Hospital. EVK: Evangelisches Krankenhaus. RUMC: Radboud university medical center. THD: Thumbay Hospital. FNA: fine needle aspiration. FNB: fine needle biopsy. PDAC: pancreatic ductal adenocarcinoma. LN: lymph node. NET: neuroendocrine tumor. GIST: gastrointestinal stroma tumor.

**Table 2:** Comparison of sample adequacy and diagnostic accuracy of the PBF needle with other FNA/B needles used.

	PBF Needle Adequate	
**FNA/B Adequate**	Yes	No
Yes	15	3
No	3	1
FNA/B not performed	2	0
	**PBF Needle Accurate**	
**FNA/B Accurate**	Yes	No
Yes	10	4
No	6	2
FNA/B not performed	1	1

**Table 3:** Comparison of sample adequacy and diagnostic accuracy of the PBF needle by lesion location.

	PBF Needle Adequate	
**Lesion Location**	Yes	No
Pancreas	7	3
Subepithelial	9	1
Lymph node	3	0
Pararectal mass	1	0
	**PBF Needle Accurate**	
**Lesion Location**	Yes	No
Pancreas	6	4
Subepithelial	7	3
Lymph node	3	0
Pararectal mass	1	0

**Table 4:** Results of the questionnaire about user experience (1 lowest; 10 highest score).

Feature	Mean	Range
Handling of the PBF needle	6.4	4–8
Puncturing of the lesion	6.6	4–8
Opening forceps in the lesion	6.0	5–7
Closing forceps in the lesion	6.0	5–7
Use in bended scope position	4.2	3–6
Visibility on US imaging	7.2	6–8
Robustness of the PBF needle	5.4	3–7
Tissue yield satisfactory	6.4	3–8

## Supplementary Materials

The following are available online at https://www.mdpi.com/article/10.3390/diagnostics11091638/s1.
