# Supplementary material for: EUS-Guided Biopsy with a Novel Puncture Biopsy Forceps Needle—Feasibility Study
Source: Diagnostics (Basel). 2021 Sep 7;11(9):1638. doi: 10.3390/diagnostics11091638 (PMC8466864; doi:10.3390/diagnostics11091638)
Supplement: Supplementary file 1 [file diagnostics-11-01638-s001.zip › diagnostics-1321656-supplementary.pdf]

Questionnaire about use of the MTW Puncture Biopsy Forceps

Score each topic on a scale from 1 – 10 (with 1 being the worst/lowest score and 10 being the best possible score). If a topic/question was not applicable in the cases you used the needle on, please write N/A.

1. Overall handling of the needle
   score 1 – 10: ___________

2. Puncturing of the lesion
   score 1 – 10: ___________

3. Opening of the biopsy forceps in the lesion
   score 1 – 10: ___________

4. Closing of the biopsy forceps in the lesion
   score 1 – 10: ___________

5. Use of the needle in bended scope position
   score 1 – 10: ___________

6. Visibility of the needle on US imaging
   score 1 – 10: ___________

7. Robustness of the needle
   score 1 – 10: ___________

8. Is the tissue yield satisfactory?
   score 1 – 10: ___________

For the questions below, please circle either 'Yes' or 'No' and explain below.

9. Did you experience a learning curve in the use of the needle?

   Yes - No

   Why Yes/No:
   ____
   ____
   ____

10. Would you keep using the needle?

    Yes - No

    Why Yes/No:
    ____
    ____
    ____

11. Would you recommend the needle to colleagues?

    Yes - No

    Why Yes/No:
    ____
    ____
    ____

12. Did you experience any problems with the needle (e.g. breaking of parts)?

    Yes - No

    Why Yes/No:
    ____
    ____
    ____

13. General remarks:
    ____
    ____
    ____
